# Communicating Effectively in Pediatric Cancer Care: Translating Evidence into Practice

**DOI:** 10.3390/children5030040

**Published:** 2018-03-11

**Authors:** Lindsay J. Blazin, Cherilyn Cecchini, Catherine Habashy, Erica C. Kaye, Justin N. Baker

**Affiliations:** 1 Department of Oncology, Division of Quality of Life and Palliative Care, St. Jude Children’s Research Hospital, Memphis, TN 38105, USA; lindsay.blazin@stjude.org (L.J.B.); erica.kaye@stjude.org (E.C.K.); 2 Department of Pediatrics, Children’s National Medical Center, Washington, DC 20010, USA; ccecchin@childrensnational.org (C.C); chabashy@childrensnational.org (C.H.)

**Keywords:** pediatric oncology, pediatric cancer, pediatric palliative care, communication

## Abstract

Effective communication is essential to the practice of pediatric oncology. Clear and empathic delivery of diagnostic and prognostic information positively impacts the ways in which patients and families cope. Honest, compassionate discussions regarding goals of care and hopes for patients approaching end of life can provide healing when other therapies have failed. Effective communication and the positive relationships it fosters also can provide comfort to families grieving the loss of a child. A robust body of evidence demonstrates the benefits of optimal communication for patients, families, and healthcare providers. This review aims to identify key communication skills that healthcare providers can employ throughout the illness journey to provide information, encourage shared decision-making, promote therapeutic alliance, and empathically address end-of-life concerns. By reviewing the relevant evidence and providing practical tips for skill development, we strive to help healthcare providers understand the value of effective communication and master these critical skills.

## 1. Introduction

Approximately 16,000 cases of cancer are diagnosed each year in children in the United States [1]. Although advances in treatment have led to remarkable gains in survival over the past century, an estimated 20% of children with cancer still die of their disease [1]. The field of pediatric palliative oncology (PPO) has emerged in response to the burden of suffering experienced by children with cancer and their families [2]. A core tenet of PPO is the use of effective communication to enhance therapeutic alliance and align the provision of medical interventions with the goals of care of the patient and family. Optimal communication in the context of pediatric oncology should begin at the time of diagnosis [3] and continue throughout the illness trajectory to enhance the therapeutic relationship, explore the hopes and goals of patients and families, and deliver care that maximizes quality of life and minimizes decisional regret.

Studies have demonstrated that patients with cancer desire effective communication with their healthcare providers (HCPs) [4,5]. Here, we will use the term healthcare providers to reference all members of a patient’s interdisciplinary team, a critical functional unit of care delivery that will be discussed in greater detail below. In this context, the National Cancer Institute and the American Society of Clinical Oncology have called for improvements in patient–provider communication [6]. Effective communication is associated with improved quality of life [7] and is essential for promoting and facilitating shared decision-making between HCPs, patients, and families [8]. Even for patients for whom no curative treatments exist, open and empathic conversations with trusted HCPs can offer hope and healing [9]. For these reasons, HCPs who strive to provide optimal care for pediatric oncology patients should prioritize high-quality communication.

Effective communication is one of the primary means through which therapeutic relationships are established and developed. Six core functions of patient–provider communication were previously identified by Epstein and Street (Table 1) [6]. This conceptual framework can aid providers in understanding the importance of patient-centered communication and gaining critical skills. A recent review article on communication in pediatric oncology is framed in part around this model and provides an overview of recent research in this field [5].

## 2. Communicating Diagnosis to Families in Distress

For many HCPs, the first opportunity for effective and empathic communication with a patient and family comes at the time of diagnostic disclosure. Here, we discuss strategies for approaching this difficult task.

The period of illness preceding cancer diagnosis is fraught with considerable psychosocial distress. Communicating diagnoses to patients and families living under such a strain can be challenging. The manner in which this information is delivered can affect patient and family adjustment to the diagnosis, both positively and negatively [10]. Because of the importance of what we say and how we say it, much thought has gone into developing communication guides for diagnostic disclosure.

One such guideline has been provided by Mack and Grier in “The Day One Talk” [3]. In this report, the authors describe the steps that HCPs can take to optimize the communication of difficult information to a family in distress (summarized in Table 2) [3].

By clearly naming the diagnosis, outlining the treatment plan, and correcting misinformation about causation, HCPs can use communication around diagnostic disclosure to foster a lasting therapeutic alliance. Although no family ever wishes to receive a cancer diagnosis, some may find relief from learning the cause of their child’s symptoms. A known diagnosis, defined treatment plan, and trusted HCP can help patients and families feel empowered to engage in treatment and prepare for the coming cancer journey.

Much of pediatric oncology is practiced in tertiary care medical centers with diverse patient populations. Excellent communicators must cultivate an awareness of and respect for the unique cultural experiences of each patient and family and work to develop a shared understanding of the essential health information. Alternative explanatory models of disease and treatment should be elicited and explored to allow the patient, family, and HCPs to develop partnerships and move toward shared decision-making. While this is a valuable topic, a full review of cross-cultural communication is beyond the scope of this article.

## 3. Prognostic Communication and the Importance of Hope

After sharing the diagnosis, HCPs should address the ways in which the disease may impact the child, including likelihood of cure and expected complications. Prognostic disclosure often provokes anxiety for HCPs and may be greeted with similar apprehension by patients and families [11]. HCPs fear that communication about prognosis may erode hope and cause distress in families who are already overwhelmed [12]. Despite these concerns, research suggests that prognostic disclosure provided by trusted HCPs in an appropriate setting confers a range of benefits to patients and families, irrespective of the nature of the information provided [12,13].

Parents of children with cancer almost unanimously wish to receive prognostic information, and most prefer to hear as much detail as is available [14]. The period following a cancer diagnosis is one of uncertainty and anxiety for patients and families. By addressing this uncertainty through clear prognostic communication, HCPs have an opportunity to help decrease anxiety around fear of the unknown. Providing accurate information may help empower families to engage in medical treatment and optimize quality of life [2].

Additionally, hope is identified as an essential part of the treatment journey by patients with cancer [15]. Although HCPs may avoid disclosing accurate prognostic information for fear of extinguishing hope [16], a growing body of evidence suggests that maintenance of hope and prognostic awareness are not mutually exclusive. A study of parents of children with cancer found that parents who receive more prognostic information experience higher communication-related hope, even when the prognosis is poor [12]. A qualitative study in a similar population found that parents view prognostic communication as difficult but necessary. Prognostic understanding may empower parents to reframe their hopes and goals [17], offering an opportunity for families to maximize quality time with their children [9]. Furthermore, parents who receive high-quality information, including detailed prognosis, self-report less decisional regret than do parents who receive less information [18].

Given that most parents desire prognostic awareness, HCPs must consider how best to communicate highly stressful and upsetting prognostic information. As with most critical conversations, HCPs should carefully plan the setting with the goal of maximizing privacy and minimizing interruptions. At the start of a conversation, patients and families should be asked about their current prognostic understanding and what additional information they wish to learn. Ask-tell-ask is a conversational technique in which HCPs elicit the specific information that a patient and family desire to know, deliver that information, and then ask the family to share what they have heard and understood. Employing this technique allows providers to gain insight into a family’s current understanding while also demonstrating a willingness to listen [19]. Simple questions such as “What have you been told?” and “What would you like to know?” can enable HCPs to tailor a conversation to the needs of a patient and family [11]. With regards to prognostic disclosure, some patients and families may wish to know numeric survival rates, while others may seek a general sense of likelihood of cure. For patients with no curative options, prognostic disclosure may center on estimations of survival length with anticipatory guidance about expected disease trajectory. Prognosis also may comprise discussions about what the disease means for a child’s future, irrespective of survival. By understanding the family’s informational needs, the HCP can be prepared to answer questions and empathically disclose additional information that may be both helpful and difficult to hear.

Despite clear communication of prognostic information, patients and families may demonstrate a nonlinear evolution of prognostic awareness. HCPs may feel frustrated when patients and families discuss future goals that do not align with a child’s realistic projected survival. However, research suggests that parents of children with cancer are able to report accurately on their child’s prognosis while simultaneously maintaining a wide range of hopes, including hope for cure [16]. HCPs should help patients and families identify and reframe new and different hopes by frequently asking the paired questions, “What are you hoping for?” and “What else are you hoping for?” By exploring these additional hopes, HCPs can broach conversation about realistic goals and encourage meaningful choices to optimize quality time for patients approaching the end of life.

Patients and families may further benefit from serial discussions about prognosis over time. HCPs may consider a longitudinal approach to prognostic disclosure in which providers facilitate prognostic awareness throughout the illness journey [20]. This approach may be particularly relevant for patients with progressive refractory disease, as they and their families struggle to reconcile their hopes for cure with the reality of incurable illness. (Table 3) [20].

In summary, effective prognostic communication is a difficult but essential task for HCPs to practice and prioritize. Patients and families who understand prognosis are empowered to make informed decisions that align with their stated goals of care. Although an engaged approach to decision-making is important for every patient, it is particularly critical for patients with no further curative options. Further communication strategies for these patients are discussed in additional detail below.

## 4. Communication with Families at the End of Life and During Bereavement

In addition to assisting patients and families with end-of-life decision-making, HCPs must also provide anticipatory guidance about the dying process. End-of-life physiology can be highly distressing for family members, particularly if inadequately explained. Expected levels of responsiveness, grimacing, agonal breathing, and other common end-of-life symptoms should be discussed in clear and specific terms prior to the onset of anticipated changes. Such explanations may help provide reassurance to parents who often feel ill-equipped to care for their children during the final stage of illness.

A critical aspect of communication around anticipatory guidance at the end of life includes discussion with families about symptom management plans and the availability of staff to ensure the highest possible level of comfort. Providing a comfortable death to children with cancer is paramount and has been identified by the National Quality Forum as a critical measure of quality care [21]. Specific symptom management plans, including contingency plans for new symptoms, should be developed in partnership with patients and parents. HCPs should be available in person or by phone to troubleshoot additional issues that may arise.

Parents of children who died of cancer strongly advocate for children to continue to receive the same level of care as they approach end of life, and many parents express fears of abandonment by HCPs when goals of care shift from cure to comfort [22]. HCPs should make every effort to assuage such fears through clear communication and actions. Evidence suggests that families who observed professionalism in the interactions between HCPs and the dying patient and who were reassured that the patient was comfortable were more satisfied with care [23]. Additionally, families that were coached on how to care for the patient were allowed adequate time to grieve after death, and those who could not overhear medical conversations outside the patient’s room reported lower levels of distress at time of patient death [23].

HCPs also can support patients and families by offering updates on clinical status. Although the timing of death can be difficult to predict, an experienced HCP should attempt to provide information regarding anticipated timeline as the patient’s end of life approaches. Providing a diagnosis of hours, days, or weeks allows families to prepare, both emotionally and pragmatically, in terms of making funeral arrangements and ensuring that loved ones can be present at the time of death. A bereavement plan of care should be developed in collaboration with the patient (when developmentally appropriate) and family during earlier advance-care planning conversations and implemented by the HCP after the patient’s death.

Following a child’s death, the role of the HCP changes yet remains highly important. The death of a child is a devastating event, and the grieving process is often intense and prolonged [24] and may include feelings of helplessness and guilt [25]. Many factors that affect parental grief are immutable, including the timing and manner of death. Some variables, however, can be moderated by HCPs in ways that influence the bereavement experience. Parents who perceive an uncaring or “too busy” attitude among staff as their child was dying report higher levels of grief near time of death. Similarly, parents who thought HCPs were being evasive were less effectively able to manage their grief. Parents who felt adequately informed and were satisfied with their child’s end of life care experienced lower levels of early grief [26].

In addition to the death of their child, many parents grieve the loss of their hospital community. Research suggests that feelings of abandonment can complicate parental grief [27]. Understandably, many bereaved parents wish to remain connected with their child’s care teams [28]. Parents of children who died of cancer specifically desire to continue relationships with the HCPs involved in their child’s care, and they identify communication with their child’s prior HCPs as an important part of their grieving process. Additionally, connecting with bereaved families may benefit HCPs by providing closure and healing. Although bereavement resources are limited in many institutions, a brief phone call to a family can be multiply beneficial by screening for complicated grief, connecting families with local resources, and, importantly, reminding the family that their child was important and is not forgotten [29].

Caring for a dying child and his/her family is a trying experience for HCPs. By optimizing comfort through effective symptom management, providing up-to-date clinical and prognostic information, and delivering empathic, patient-centered care, HCPs can support patients and families through this devastating event.

## 5. Cultivating Therapeutic Alliance in the Patient–Provider Relationship

Therapeutic alliance encompasses the personal bond and shared therapeutic goals among the patient, family, and HCP [30]. Establishment of a therapeutic alliance begins at the first meeting and further develops over time. Optimal communication strengthens the therapeutic alliance, providing HCPs with necessary credibility when difficult decisions must be made.

Stronger therapeutic alliance between patients and physicians is associated with improvements in patient and family psychosocial outcomes, including illness coping, quality of life, and treatment satisfaction [31]. Additionally, a strong alliance is associated with increases in perceived social support, decreased illness-related grief among adolescents and young adults with cancer, and improvements in treatment adherence [31]. The latter finding is particularly salient to note, as rates of nonadherence in adolescents with cancer approach 60% and are associated with poor clinical outcomes.

Relationships between patients and families and the care team become increasingly important in the context of refractory, progressive, or relapsed disease. Although HCPs may worry that frank discussions about prognosis may weaken or damage the patient–provider relationship [12], open conversations about end-of-life care do not adversely impact therapeutic alliance [32]. On the contrary, a strong therapeutic alliance is associated with greater emotional acceptance of incurable illness in patients with cancer [32]. By devoting time and effort towards establishing and developing therapeutic alliance, HCPs can help empower patients and families to confront their cancer diagnoses and reframe their hopes and goals for the future.

Furthermore, the benefits of a strong therapeutic alliance extend beyond the patient. Caregivers of patients with cancer who perceive a supportive alliance between the patient and HCP self-report decreased role limitation, enhanced social function, and improved physical and psychological health [33]. Importantly, caregiver benefits persist after the death of the patient [33].

Given the myriad of benefits for patients and families, establishing a therapeutic alliance should be a priority for HCPs. Many of the skills detailed above can serve to develop and strengthen the therapeutic alliance. The International Society for Paediatric Oncology (SIOP) developed guidelines to assist HCPs in developing a therapeutic alliance with families (Table 4) [34].

**Figure 1:**
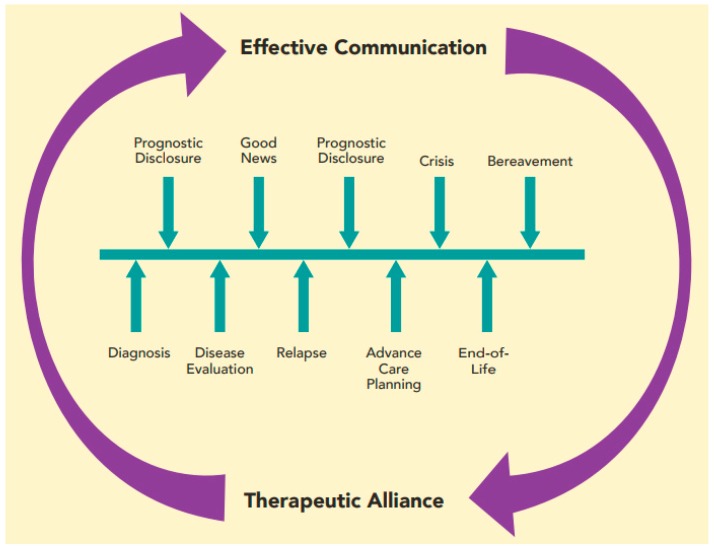
Diagram of the cyclic, reinforcing relationship between effective communication and therapeutic alliance throughout illness trajectory.

Establishment of a strong therapeutic alliance with HCPs benefits patients and families. Effective, empathic communication is one of the primary means by which this alliance is forged. Over time, the strength of the therapeutic bond with a family allows HCPs to discuss more difficult topics. If done well, these challenging conversations serve to further deepen the therapeutic alliance and create space for future discussions. Effective communication deepens the therapeutic alliance, which, in turn, allows for more open communication. In this way, these two concepts are mutually reinforcing and foster greater connections between patients, families, and HCPs over time (Figure 1).

## 6. Involving the Child

Determining how and when to involve pediatric patients in medical conversations is a critical skill for pediatric oncology HCPs to master. Most childhood cancer survivors and their families believe that a cancer diagnosis should be discussed with a child early in the disease course [35]. Effectively including children in medical conversations requires excellent communication skills, strong alliance with parents, and a thorough grasp of developmentally-appropriate communication strategies (Table 5) [36]. Though challenging, it is important that HCPs engage children in conversations about their health to ensure that their unique questions, fears, and uncertainties are addressed [37].

Any plan to involve a child in medical conversations should be developed with parents. Out of a desire to protect their child, some parents are hesitant to disclose illness information. In these circumstances, HCPs should broach conversations by asking parents to share what they believe their child already knows. Even young patients can understand serious illness and participate in medical decisions as long as conversations are led in an age-appropriate manner [38]. Furthermore, children often perceive the seriousness of their illness prior to hearing formal disclosure by family or HCPs. By helping parents recognize the level of their child’s illness awareness, HCPs can empower parents to explore difficult topics with their child.

A majority of chronically ill adolescents wish to be involved in their medical decision-making [39]. The degree of engagement varies, with many adolescents preferring to play an active role in their medical decision-making [40]. Even those that prefer less active roles still desire to be informed about their health [40]. HCPs should make every effort to involve adolescent patients in medical conversations, to the level that is desired by the adolescent and family. Even participation in relatively minor care conversations may allow adolescent patients to regain control and build trust with HCPs [40].

The literature also supports the involvement of adolescent patients in advance care planning. Family-centered advance care planning (ACP) elicits input from adolescent patients and their parents and is associated with improved congruence of end-of-life care with the stated goals of patients and families [41]. Patients and families who participate in family-centered ACP find the conversations to be difficult but worthwhile [42], report a greater understanding of end-of-life wishes, and are more likely to receive early palliative care [43]. Importantly, all of these studies used structured conversation guides to facilitate discussions. Evidence-based conversation guides, such as Voicing My CHOICES (available from Aging With Dignity [44]), are available to assist HCPs who wish to enter into these difficult but necessary discussions with patients facing life-limiting illnesses.

The benefits of engaging patients in medical conversations persist even when there is no longer chance for cure. Parents who involve their children in discussions about prognosis and impending death generally do not regret doing so [45,46]. Disclosing to a family that there are no further curative options for their child’s disease is challenging. HCPs facilitating these conversations should be mindful of the language they choose and avoid terminology that may seem dismissive or offensive (Table 6) [47]. Other team members including child life specialists and spiritual care providers may be particularly helpful for navigating these challenging discussions.

In summary, facilitating patient involvement in their own care is one of the essential functions of HCPs. While challenging, there is a growing body of evidence that suggests involving pediatric patients in medical conversation in age-appropriate ways has benefits for both patients and families.

## 7. Communicating with Siblings and the Extended Family

In addition to interacting directly with patients and parents, HCPs also play an important role in facilitating communication within the larger family unit [48]. In particular, HCP communication with siblings of children with cancer can considerably affect how a family adjusts to the diagnosis [49]. The families of children who die from cancer are also impacted by their interactions with HCPs. Bereaved parents appreciate HCPs who engage the family in conversations about care decisions [24]. Siblings who receive adequate communication at the end of their sibling’s life have lower levels of long-term maladjustment. Importantly, the inverse is also true: siblings who did not perceive satisfactory communication around their sibling’s death subsequently report higher levels of anxiety later in life [50].

In communication with the family unit, HCPs must remain mindful of parent and sibling preferences as well as relevant spiritual and cultural beliefs or practices. HCPs can encourage parents to openly discuss a patient’s illness with his/her siblings while respecting parental wishes and beliefs related to information sharing. Families who choose to discuss a patient’s illness with siblings and other family members may require support from trusted HCPs. It may be beneficial for HCPs to meet with parents before discussions with the extended family to answer questions and develop strategies for sharing medical information. Some families may benefit from having the HCP available during the conversation to answer questions.

HCPs and parents can employ several strategies when conveying truths about a patient’s disease with the extended family. HCPs should state that they are available to assist parents with these conversations and will respect their decisions regarding what information to disclose and to whom. HCPs should either be present during the conversation or be available at a set time to answer questions from the extended family. Remind parents that, though they can be difficult to say, words like “death,” “dying,” and “cancer” should be used to make sure family members understand the clinical situation. Parents may be worried about responding to emotions or about their own emotional reactions. HCPs can reassure that a wide range of emotional responses to this kind of information is normal. Parents may find phrases like “Mommy is crying because she is sad that (child’s name) is sick” helpful in these situations. For parents discussing a cancer diagnosis with the patient’s siblings, additional effort should be made to ensure information is discussed in a developmentally appropriate way.

In summary, a cancer diagnosis in one child affects the entire family. By facilitating communication with and among all family members, HCPs can help family members adjust to their new roles, process their emotions, interact appropriately with the patient, and cope with the aftermath of the death of a child.

## 8. An Interdisciplinary Team Approach to Communication

To best support the physical, emotional, and spiritual needs of patients and families, pediatric palliative oncology care should be provided through an interdisciplinary team (IDT) [51]. This team may be composed of physicians, nurses, advance practice clinicians, social workers, chaplains, child life specialists, and other psychosocial support staff. IDT members must communicate effectively with one another and with the patient and family in order to ensure provision of optimal medical, psychosocial, and spiritual care.

Individual members of the IDT should meet with the patient and family to provide targeted support of their physical, emotional, and spiritual needs. The IDT should meet at regular intervals so that insights gleaned from individual conversations can be shared with the entire team [52]. The primary goal of the IDT is to integrate the clinical perspectives and expertise of a diverse group of providers to develop a holistic care plan that supports the expressed and perceived needs of the patient and family.

Clinicians who participate in the IDT meeting should be cognizant of the common phenomenon in which patients’ biomedical needs are reviewed in detail with relatively little attention given to spiritual and emotional needs [52]. HCPs should guard against the biomedical bias by conducting IDT meetings with a structured format that ensures an appropriate balance of physical, emotional, and spiritual issues. Ideally, one designated clinician should lead the meeting to facilitate efficient discussion and transitions. This clinician should share a one-line medical summary for the patient then invite other interdisciplinary colleagues to provide salient information to enhance the team’s collective understanding and facilitate improved care coordination/planning for the patient and family.

**Figure 2:**
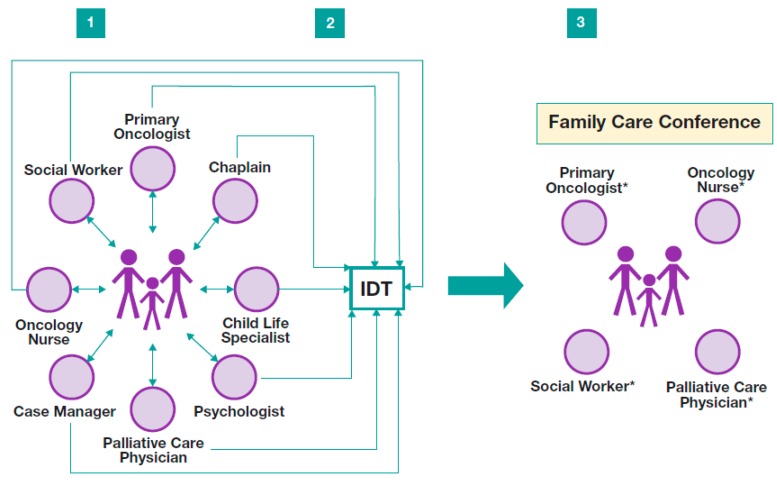
Flowchart demonstrating ideal collaborative efforts to promote effective communication of comprehensive care plans. Panel **1** illustrates individual interdisciplinary team (IDT) members meeting with patient and family; Panel **2** illustrates the IDT meeting; and Panel **3** illustrates the family care conference conducted based on recommendations from the IDT meeting. IDT members will be chosen to participate in the family conference based on the information to be discussed and the needs of the patient and family.

Often, information discussed during an IDT meeting needs to be shared with patients and families. Typically, this transfer of information is best facilitated through a family conference. Figure 2 depicts the typical clinical progression from individual HCP conversations to IDT meeting to family conference [53].

A family conference can be loosely defined to include any planned meeting between IDT members, patients, and family members in which discussions of the patient’s clinical status, illness trajectory, treatment plan, prognosis, goals of care, or disposition planning occur [54]. HCPs may identify the need for a family conference in the setting of a change in clinical status, in anticipation of future decision making, prior to an upcoming transition, in the context of fragmented or ineffective communication, or in response to moral distress or ethical concerns raised by the care team or family [55].

While the objectives of individual family conferences vary based on the clinical circumstances and key stakeholders unique to each case, the primary goal underpinning the agenda for all family conferences should be to ensure that the patient, family, and HCPs have a shared understanding of the illness trajectory, appropriate treatment plan, and overall goals of care. Prior to initiating a family conference, the IDT should discuss and identify what information should be conveyed to the patient and family. Based on this, the team then may elect certain representatives to lead and attend the family conference.

The optimal time to schedule a family conference is when the patient’s clinical condition is reasonably stable and the patient and/or family are already aware of the illness and its implications [56]. However, often family conferences occur in circumstances of extremis, in which the patient is acutely ill or worsening and/or families are unaware of the gravity of the situation. HCPs should strive to coordinate family conferences prior to catastrophic events, yet they should not shy away from scheduling conferences even during times of extreme stress.

An effective family conference requires preparation, particularly from the HCP that will be leading the conference. It is critical that HCPs have a comprehensive understanding of the patient’s medical condition, realistic treatment options, and goals of care. The patient and family should be given adequate notice regarding the timing of the conference and a time and location should be chosen that accommodates the family and all key team members. In advance of the meeting, patients and families should be encouraged to create an agenda or question list to be addressed during the meeting.

It is also important for HCPs to ask the patient and family who would be most helpful to have in attendance at the conference, including members of the IDT, other members of the medical team, and family members. The patient may or may not attend the conference, depending on age/developmental stage, clinical status, and personal or family preferences. If the patient does not attend, arrangements should be made in advance for an appropriate family member or clinician (e.g., a child life specialist, music therapist, or other known and trusted HCP) to remain with the patient if desired by the child or family.

Although family conferences occur frequently within the pediatric oncology setting, the majority of HCPs do not receive formal training on how to effectively lead or communicate in these conferences [57,58]. The following discussion outlines general recommendations for facilitating a family conference, including guidance regarding language options for HCPs to use.

At the specified time, all attendees should gather and be seated. The HCP designated to lead the conference should facilitate introductions of all team members and family members in attendance. The conference leader should then review the agenda for the meeting and ensure concurrence between family and providers. Prior to sharing medical information, the conference leader should ask the patient and family a few open-ended questions to elicit their understanding of the clinical situation. This may include statements like, “Tell me what you’ve heard the doctors say” or “What have you been told about how (the patient) is doing?” The responses of the patient and family to these questions should shape the structure and content of the remainder of the conference.

The meeting leader should provide a concise summary of the relevant medical facts and an overview of therapeutic options, if relevant, taking care to only include plans considered reasonable and feasible for the patient [55]. The leader should also facilitate the sharing of information from subspecialized experts in attendance, encouraging each clinician to provide information and then summarizing and integrating the input into the larger clinical picture. Depending on case-specific factors, the conference leader’s role may focus more on facilitating the transfer of information between other experts and the family, as opposed to delivering information directly. An important responsibility of the conference leader is to make statements related to the most likely disease trajectory and outcome, providing anticipatory guidance even in the context of prognostic uncertainty.

During each phase of a family conference, HCPs must strive to provide clear information devoid of medical jargon, using language that accurately and frankly describes the patient’s current status and most likely anticipated outcome. Questions from the patient and family must be answered honestly and concisely, ideally in 2–3 sentences, followed by a significant pause to allow the family to process the information. Regardless of the prognosis or treatment options offered, it is imperative to provide reassurance that the patient will continue receiving excellent, attentive care. Statements that affirm that the IDT will remain highly engaged in the patient’s management and strive to provide care that aligns with the goals of the patient and family can be helpful, and positive language that emphasizes what CAN and WILL be done is preferable to language that describes what cannot or will not be done. For example, saying, “We will continue to do everything possible to ensure that (the patient) lives as well as possible for as long as possible” or “We will continue to fight to do everything we can so that (the patient) is as comfortable as possible” can help the patient and family know that their team continues to prioritize their needs and will not abandon them in the face of progressive illness.

Additionally, HCPs should strive to maintain a respectful environment wherein all conference attendees are able to offer insights and interruptions are minimized [56]. Patients and family members should be encouraged throughout the conference to share their hopes, fears, and concerns so that these may be addressed as specifically as possible by the IDT members in attendance. Questions such as “What are you most worried about?” also offer helpful ways for HCPs to transition into conversations about prognosis and goals of care. Sitting in silence can be another effective way to elicit difficult emotions and concerns; HCPs should be reminded before the conference of the value of sitting quietly and not filling the silence, but rather deferring to the patient, family, and meeting leader to determine the duration of pauses.

Lastly, at the conclusion of a conference, patients and families should be provided with clear and specific information about the next time they will meet and how to reach the team if new questions or concerns arise in the interim. Within 24 h after the conference, at least one member of the IDT should follow up with the family to help them process the information and address any lingering questions or concerns. An overview of the conference should also be documented systematically and clearly in the patient’s medical record, with copies sent to all IDT members to ensure that the team moves forward collaboratively and communicates with the patient and family in ways that align with the information shared during the family conference.

In summary, comprehensive care of pediatric oncology patients and their families in the modern health care system extends beyond the capabilities of any one clinician. The complexity of patient medical needs is mirrored by multifaceted and overlapping communication lines between the patient and family and all participating HCPs. IDT meetings and family conferences are two ways in which HCPs can coordinate the efforts of a variety of providers and collaborate with patients and families to optimize the provision of holistic, goal-concordant care.

## 9. Conclusions

Effective communication is an essential skill in the practice of PPO. Mastery of effective communication enables HCPs to expertly disclose diagnoses, facilitate the development of prognostic awareness, navigate advance care planning discussions, identify goals of care, provide comfort at the end of life, and support bereaved families during the difficult grief journey. Improving skills with regards to development of therapeutic alliance, involving pediatric patients in medical discussions, communicating with the whole family unit, and collaborative dialogue in the context of an IDT approach allows HCPs to provide optimal care to patients and families. A large body of evidence demonstrates the benefits of effective communication on patient and family experiences and outcomes. HCPs who strive to provide excellent, whole-person care must devote time and attention to the development of these critical communication skills.

## Figures and Tables

**Table 1:** Six core functions of patient–provider communication.

Functions	Communication Methods
Responding to emotions	Evaluate and appraise distress Offer validation, empathy, and support
Exchanging information	Identify depth of information the patient or caregiver desires
	Acknowledge the abundance of information available online
	Consider findings presented without seeming dismissive
Making decisions	Partner with patient and family to identify goals of care
	Align treatment plan with stated goals
Fostering healing relationships	Develop mutual trust, understanding, and commitment
	Clarify roles and expectations of patient and provider
Enabling self-management	Encourage active engagement in all aspects of care
	Invite discussion and questions from patients and families
Managing uncertainty	Recognize limitations in knowledge
	Name uncertainties and address associated fears

**Table 2:** A structured approach to diagnosis disclosure.

Components	Key Steps	Examples
Prepare the setting	Quiet location	
	All desired parties present and seated	
	Minimize interruptions	
Elicit understanding	One HCP takes the lead, asks family to describe their current understanding	“What have you heard so far about what is going on?”
Provide “warning shot”		“I’m afraid we have difficult news to discuss.”
		“Unfortunately, the scans didn’t show what we hoped.”
Give the diagnosis	Use clear language	“Your child has leukemia, which is a kind of cancer.”
	Avoid euphemisms	
	Use the word cancer	
Pause	Stop speaking	
	Allow the family to process information	
	Elicit questions	
Discuss treatment	Discuss expected location and duration of treatment	“We will use a combination of surgery followed by medicines called chemotherapy to treat the cancer. Most of the chemotherapy will be given during inpatient hospitalizations lasting 3–5 days. Overall, treatment will last for about 6 months.”
	Explain different modalities	
	Provide alternative options	
Pause	Stop speaking	
	Allow the family to process information	
	Elicit questions	
Define goals of therapy	Provide clear, honest communication regarding curative intent	“The goal of therapy is to cure your child’s cancer.”
		“Unfortunately, there is no cure for this cancer at this time. The goal of treatment will be to minimize symptoms, improve quality of life, and prolong life.”
Pause	Stop speaking	
	Allow the family to process information	
	Elicit questions	
Address causation	If accurate, clearly state that cancer was not preventable	“We don’t know what causes this kind of cancer, but we know that there is nothing that you or your child did to cause it. You did the right thing by bringing your child in when you did.”
	Dispel concerns that cancer resulted from something child or family did or did not do	
Summarize key points	Restate the diagnosis, goals of therapy, and discussion of causation	“For today, what I want you to understand is that your child has cancer. We plan to treat with chemotherapy and the goal of treatment is cure. There is nothing you or your child could have done to prevent this and this is not your fault.”
Conclude conversation	Offer reassurance that information will be discussed again at future visits	“We will discuss all of this information again, so don’t worry if you can’t remember everything. I will see you in clinic again tomorrow afternoon. If you have any concerns before then, you can always call the clinic at...”
	Plan for next visit	
	Provide contact information for urgent issues	

**Table 3:** Cultivating prognostic awareness over time.

**Step 1. Assess understanding of disease and prognosis**	
What have you been told about your/your child’s disease/prognosis? What is your sense of what the future holds? How worried are you? What has you the most worried?	
**Step 2. Facilitate development of prognostic awareness by imagining poorer health**	
Have you ever thought about what it might be like if you/your child got sicker? It might be good to think about what might happen if you/your child got sicker. It is good to be prepared in case that does happen.	
**Step 3. Assess response and consider urgency of need to deliver prognostic information**	
*If the patient is ambivalent about prognostic discussion and disease is stable:*	Delay giving prognostic information Repeat steps 1–3 over time to cultivate prognostic awareness
*If the patient is ambivalent about prognosis discussion but disease is worsening:*	Align with the patient Name the dilemma: “It seems like it is hard for you to talk about the possibility that you/your child might get sicker.”
*If the patient is ready to discuss prognosis, regardless of disease state:*	Deliver prognostic information Ask-tell-ask: Find out what the patient wants to know, deliver information, then ask what they make of it Pair hope and worry: “I hope you will feel good for a long time, but I am worried because your scans look much worse.”

Adapted with permission from [20].

**Table 4:** Guidelines for developing therapeutic alliance with patients and families.

Treat all members of the interdisciplinary team with professionalism and respect for their expertise Invite patients and families to be members of the medical team and recognize their unique skills and contributions Elicit needs and preferences from patients and families; avoid assumptions Provide education about diagnosis and treatment; ensure understanding Recognize the burdens associated with diagnosis; empathize with frustrations related to medical bureaucracy Meet regularly with the interdisciplinary team to discuss the psychosocial wellbeing of patients and families and strategize approaches to improve this wellbeing Support pediatric patients’ needs for autonomy and encourage them to take control of appropriate aspects of care

**Table 5:** Communication goals by patient age.

Age	Communication Goals
Infants	Soothe and relieve distress Show care through gentle touch
Toddlers	Includes prior goals for infants Elicit bothersome symptoms Validate emotional experiences
School-Aged Children	Includes prior goals for infants and toddlers Obtain information needed to diagnose and treat Encourage cooperation and adherence with recommendations Educate about disease Demonstrate respect for individual choice and voice
Adolescents	Includes prior goals for infants, toddlers, and school-aged children Recruit for discussions about goals of care and medical decision-making Elicit hopes, worries, fears

**Table 6:** Communication in the context of progressive or refractory disease.

Potential Pitfalls	Phrases to Avoid	Alternative Phrases
Placing burden of understanding on the family	“Do you understand what I’ve told you?”	“Does this make sense?”“Tell me what you’ve been hearing from the team.”
Appearing to give up	“There is nothing more we can do.”	“I wish we had a treatment to cure this disease. We will continue to do everything in our power to care for (child’s name).”
Claiming understanding	“I understand how you feel.”	“I can’t imagine how you must be feeling. I wish we had better news. What might be helpful for you right now?”
Using clichés, emphasizing the positives	“This will make you a better/stronger person.”	“May I just sit with you for a while?”
